# Evaluation of therapeutic efficacy of Emustil drops for ocular discomfort and tear film osmolarity using different treatment management modes under dry environmental conditions

**DOI:** 10.1186/s12886-024-03390-0

**Published:** 2024-03-25

**Authors:** Ali Abusharha, E. Ian Pearce, Tayyaba Afsar, Suhail Razak

**Affiliations:** 1 Department of Optometry, College of Applied Medical Sciences, King Saud University, Riyadh, Saudi Arabia; 2 Glasgow Caledonian University, 70 Cowcaddence Road, G4 0BA Glasgow, UK; 3 Department of Community Health Sciences, College of Applied Medical Sciences, King Saud University, Riyadh, Saudi Arabia

**Keywords:** Oil in water emulsion, Ocular symptoms, Tear production, Tear osmolarity, Ocular surface temperature, Dry environment, Low humidity

## Abstract

**Background:**

We aimed to check the efficacy of Emustil (oil in water emulsion) drops on tear film index and ocular surface dynamics in dry environments through protection and relief treatment modalities.

**Methods:**

The subjects were exposed to a dry environment using a Controlled Environment Chamber (CEC) where the relative humidity (RH) was 5% and the temperature was 21 °C and screened for ocular symptoms, tear osmolarity, ocular surface temperature (OST) and tear production using ocular Surface Disease Index questionnaire (OSDI), OcuSense TearLab Osmometer, FLIR System ThermaCAM P620 and Schirmer strips/phenol red test respectively. Tear production was calculated by the Tear Function Index test (TFI).

**Results:**

The mean tear film osmolarity decreased significantly from 296.8 mOsm/l at 40% RH to 291 mOsm/l at 5%. (*p* = 0.01). Instillation of Emustil resulted in a significant increase in tear osmolarity in the relief method compared with osmolarity seen at 5% RH when no drop was used. The mean PRT value decreased from 26 ± 9 in normal conditions (40% RH) to 22 ± 4 mm in dry conditions (5% RH). Emustil drops did not induce any significant change in tear production in the PRT test. No significant change was found in OST following exposure to 5% RH. OST did not show a statistically significant change with the emulsion when used for relief (*p* > 0.05). The mean score of ocular discomfort observed was 70 at 5% RH. Still, the instillation of the oil-in-water emulsion (Emustil) resulted in a noticeable decrease in visual discomfort to 37 (*p* = 0.00) in protection and 59 in relief (*p* = 0.05). Emustil drops substantially improved tear film parameters under a desiccating environment, however, tear film parameters respond differently to the management modalities. In the protection method, tear film osmolarity was protected against a dry environment, while in the relief mode, tear production was improved.

**Conclusion:**

CEC allows for a thorough evaluation of tear film parameters and dry eye treatment protocols in labs, providing greater confidence when applying them to patients. In addition, our study showed that Emustil not only provides protection and relief for dry eyes but also helps to maintain ocular homeostasis in desiccating environments. This indicates a promising potential for improving dry eye treatment protocols.

**Supplementary Information:**

The online version contains supplementary material available at 10.1186/s12886-024-03390-0.

## Key points

***What is known***

• Ocular surface discomfort experienced in dry environment conditions could result from the physical alteration of the tear film dynamics. Artificial tears and lubricants are commonly used to treat the signs and symptoms of dry eye, however, the main drawback of most of these formulations is the short retention time as the solution can easily drain via the lacrimal drainage system.

• To analyse the efficacy of eye drops various experimental methodologies have been developed. These methods have many limitations including a lack of accurate control of the relative humidity and exposing the eye to an airflow that may have induced an additional effect on reflex lacrimation.

***What is new***

• The effect of the environment on the tear film dynamics and efficacy of oil in water emulsion has been investigated by several studies, however, they were carried out under normal environmental conditions. As yet, no studies have been carried out to examine the effect of immediate acute effect of oil-in-water when used under adverse conditions in normal healthy subjects or dry eye patients.

• A new technique of inducing signs and symptoms of dry eye by exposing normal subjects to a desiccating environment [5% relative humidity (RH)] was utilized using a controlled environmental chamber (CEC). This method allowed full control of RH and to non-invasively expose and observe the tear film parameters.

• The efficiency of an oil-in-water formulation to protect and relieve the changes of tear film parameters that are caused by exposure to ultra-dry environmental conditions (5% RH) was examined. We assessed the ability of Emustil to maintain the homeostatic integrity of the ocular surface during exposure to dry condition. and assessed tear parameters including tear osmolarity, tear production and its relationship with ocular discomfort.

• Our goal was to avoid as many drawbacks as possible from previous works. Using a controlled environmental chamber allowed full control of RH without any direct contact with the eye or the skin around it or any airflow. This method allowed us to non-invasively expose and observe the tear film at different time points.

## Background

The ocular surface necessitates a stable tear film to sustain its functions; adequate production, retention, and balanced elimination of tears are essential for this process. Any variation of these mechanisms can consequences in the complaint of dry eye [1]. Ocular discomfort that is experienced in dry environment conditions could result from the physical alteration of the tear film parameters such as increased evaporation rate [2, 3], poor tear film stability [4, 5] and decreased tear film production [6]. Dry eye disease (DED) is a recurring ocular surface ailment concomitant with amplified tear osmolarity and mild to serious ocular surface irritability and inflammation [7]. A distinct biophysical mechanism that captures the equilibrium of inputs and outputs from the tear film dynamics is tear osmolarity. Osmolarity is the end product of deviations in tear dynamics and is considered a crucial mechanism underlying ocular surface impairment in DED [8]. Normal homeostasis necessitates regulated tear flow, the primary driver of which is osmolarity [9]. It has been proposed that tear hyperosmolarity is the primary reason for discomfort, ocular surface impairment, and inflammation in dry eyes and may serve as the single best objective marker for DED assessment [10]. However, the benefits of measuring tear osmolarity in the diagnosis of DED have been undermined by the difficulties of its measurement [11]. Most often DED inspection and/or medication is not executed until a patient is symptomatic. Sullivan and colleagues highlighted that 43% of asymptomatic patients had clinical signs of dry eye (DE) and if left untreated, it can significantly impact a person’s vision and quality of life [12]. The ocular surface temperature has been found to correlate well with many ocular diseases such as dry eye, ophthalmic postherpetic neuralgia, carotid artery stenosis, unilateral exophthalmos, Graves ophthalmopathy, postsurgical intraocular inflammation, bulbar conjunctival hyperemia, diabetic retinopathy, central retinal vein occlusion, glaucoma and inflammation of the lacrimal drainage system [13].

Many formulations have been invented to manage the complications of DE. The initial oil-in-water emulsion was applied in a recipe with an anti-inflammatory mediator to make up a cyclosporine eye drop (Restasis). Cyclosporine in oil emulsion was reported to be effective in the management of dry eye signs and symptoms. Sall et al. reported that it took 4 months before the full formulation with cyclosporine significantly outperformed the vehicle [14]. This vehicle was then modified to an oil-in-water emulsion with no anti-inflammatory agents included and marked as an over-the-counter artificial tear (Refresh Endura, Allergan, Irvine, USA) [15]. Similarly, castor oil emulsion has been shown to decrease ocular symptoms significantly [16]. It has been suggested that castor oil emulsion could improve ocular comfort because of its ability to improve lipid layer spread and long relative residence time (up to 4 h) [16]. The same study has shown an increase in the level of fatty acid (x2) and triglycerides (x10) in tear samples four hours following instillation of the oil-in-water [16]. Several other lipid-based eye drops have been developed such as Soothe (Alimera, Alpharetta, USA) and Emustil (SIFI, Catania, Italy) [17]. Previously three over-the-counter tear supplements were tested and Emustil emulsion was found to be significantly effective in improving tear film evaporation, tear osmolarity and corneal staining in DE patients [18]. Frequently many people are exposed to adverse environments or artificial environments. The effect of the surroundings on the tear film dynamics and efficacy of oil in water emulsion has been examined previously, however, most of the investigations were conducted under normal environmental conditions. So far, no examinations have been carried out to analyse the immediate effect of the acute effect of oil-in-water when used under adverse conditions in normal healthy subjects or DE patients. Therefore, we intended to observe the efficiency of an oil-in-water formulation to protect and relieve the changes of tear film parameters that ensued upon exposure to excessive dry environmental conditions (5% RH). In the current work as many drawbacks as possible of previous works were avoided. Using a controlled environmental chamber allowed full control of RH without any direct contact with the eye or the skin around it or any airflow. This method allowed us to non-invasively expose and observe the tear film at different time points. Also, various tear film parameters were observed under a dry environment over many time points. This helped to investigate the interrelationship between each of the tear film parameters.

## Methodology

## Materials and methods

### Participants

All subjects who participated in this study were male because the recruitment process was done by word of mouth to friends and colleagues. On the initial visit, all subjects were screened for tear production, stability and ocular symptoms. 12 males (mean age 34.0 ± 7.0 years) were enrolled in the current examination to further observe tear film parameters under varied environmental conditions.

### Inclusion and exclusion criteria

The subjects enrolled in testing fulfilled the inclusion criteria stated below:

- < 12 score in Ocular surface disease index (OSDI) [19].
- Schirmer test > 10 mm in 5 min.

Participants with a history of ocular surgery, ocular infection or who wear contact lenses were excluded from the investigation. The basal measurements of tear film parameters at normal conditions (40%RH/21°C) were carried out during the screening visit.

### Parameter analyzed

A Schirmer test was done to measure tear production [20]. Ocular Surface Disease Index questionnaire (OSDI) was utilized to assess ocular symptoms [19]. All study procedures were approved by the Glasgow Caledonian University Research Ethics Committee. Signed consent was obtained from all subjects before participating in this study. Relative humidity (RH) and temperature of laboratory surroundings were maintained using a Controlled Environmental Chamber (CEC). Detailed procedure is described in our previous report [21]. Two environmental conditions were maintained in the CEC:

1. Normal environment 40% RH at 21°C.
2. Desiccating environment 5% RH at 21°C.

### Study design

The oil-in-water emulsion used in this study was Emustil Natural eye drop emulsion (Moorfield Pharmaceutical, London, UK). Preservative-free single-dose units of Emustil were used. The Emustil drops are developed using 7% Soyabean oil and 3% Natural phospholipids (derived from egg yolk), hence containing both polar and non-polar lipids [18]. We anticipated evaluating the efficacy of Emustil drops in protection (before exposure) or relief (post-exposure) treatment schedules under dry environmental conditions and, observed its outcome on human tear film parameters.

### Scheme of treatment

The participants were allocated incoherently into two groups to study the influence of dry surroundings on the ocular surface using the protection and relief treatment plans. In the relief treatment strategy, Emustil drops were instilled after 15 min of exposure to 5% RH (dry environment) to determine if any relief was experienced by the subjects. Whereas in the protection method, the drop was instilled before exposure to dry conditions, and then the tear film parameters were assessed after exposure to 5% RH for 15 min. These two treatment protocols were described in detail in our previous reports [21, 22].

### Parameter analysed

Specific tear physiology tests were performed to diagnose the ocular condition/symptoms. Tear film osmolarity was assessed using an OcueSense TearLab [23]. Due to a shortage of Tear Function Index (TFI) supply caused by the manufacturer, tear production was measured in this study using Phenol Red Thread (PRT) [24]. Ocular surface temperature (OST) was monitored in this study by using a FLIR System ThermaCAM P620 [25]. In addition to tear physiology examination, ocular comfort was assessed in this study using a visual analogue scale questionnaire [26]. Brief details of each procedure are described in the below sections.

### Ocular surface disease index questionnaire (OSDI)

This questionnaire was designed by Allergan Inc. (Irvine, California, USA) to assess ocular surface symptoms [19]. OSDI take account of three major categories of symptoms. These include.

(i) Ocular symptoms i.e. grittiness, sensitivity to light, soreness, blurred and poor vision.
(ii) Vision function i.e. driving at night, reading, working with computers and watching TV.
(iii) Environment-related symptoms i.e. itchy in windy conditions, in air-conditioned rooms or in dry places [19].

The OSDI has a total score of 100 points. A person with a score of fewer than 12 points has a healthy tear function index [5]. The OSDI also offers a classification of dry eye disease progression based on the overall OSDI score (Table 1) [11, 12].

**Table 1:** Classification of Dry Eye (DE) progression based on the complete OSDI score

OSDI score	Diagnosis
0–12	healthy
13–22	Mild DE symptoms
23–32	Moderate DE symptoms
33–100	Serious DE symptoms

### Infrared thermography of ocular surface

**Fig. 1:**
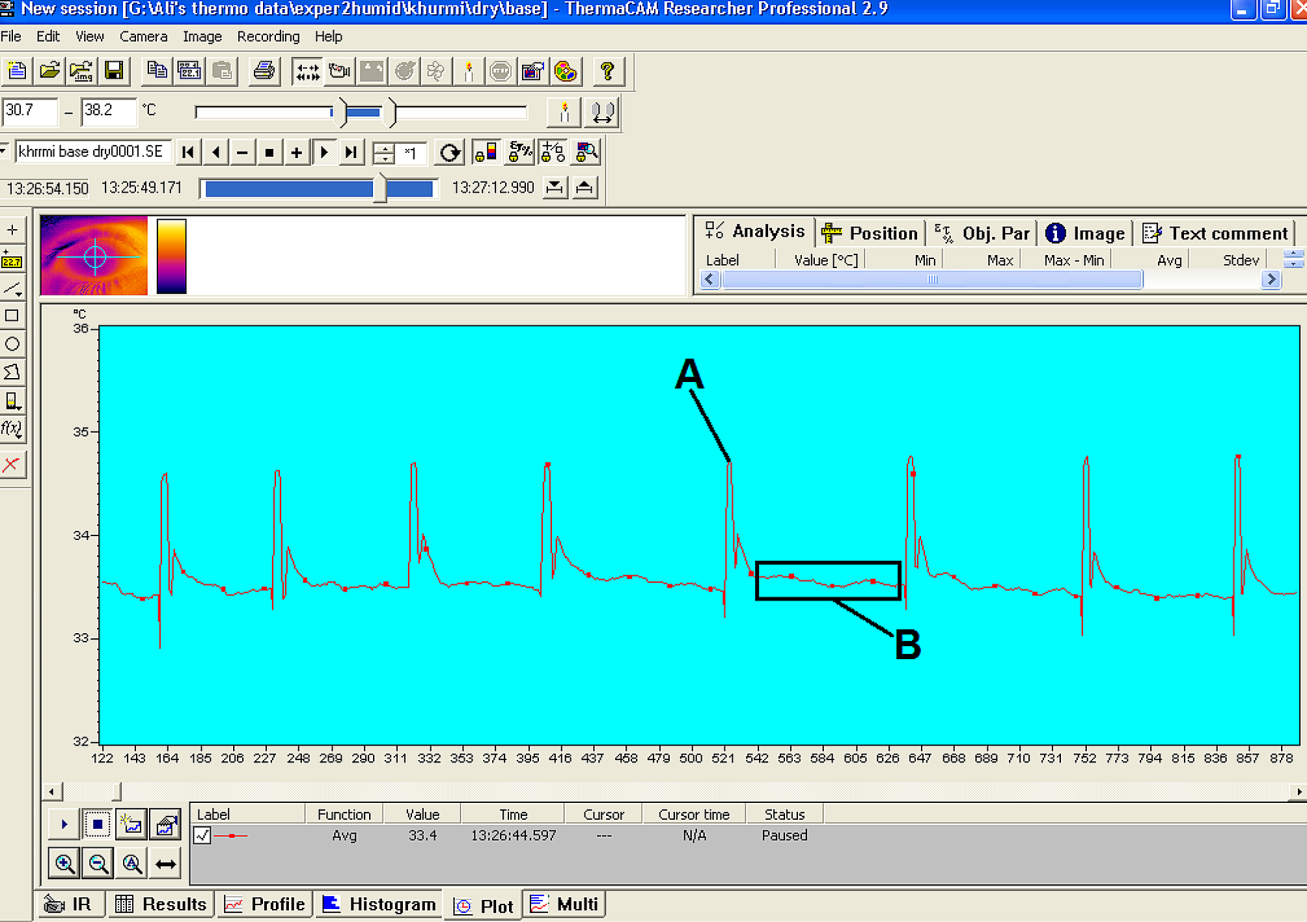
Figure shows a screenshot of ThemaCAM Researcher Professional showing the temporal change in OST with blinking (**A**) and the selected data for analysis (**B**). Data obtained during and immediately post-blink was excluded

To monitor changes in ocular surface temperature without being invasive, a FLIR System ThermaCAM P620 (FLIR Systems, Surrey, UK) was operated in the current investigation [25]. The self-calibrating camera is equipped with a high-definition detector (focal plane array, 640 × 480 pixels) that is thermally sensitive to ± 0.06ºC and capable of sensing temperatures ranging between − 40 and + 500 ºC. A close-up lens with a spatial resolution of 50 μm was attached to the camera to provide a clear and focused image of the ocular surface. The emissivity of the camera was set at 0.98. The temperature of the entire eye surface, including the upper and lower eyelids, was noted uninterruptedly for one minute at a 30 Hz frequency rate (Fig. 1). To analyze the data, the real-time image was recalled and viewed with a colour scale employing ThemaCAM Researcher Professional version 2.9 software (FLIR Systems, Surrey, UK).

### TearLab osmometer

In the current investigation, an Ocusense Tear Lab Osmometer (OcuSense, Inc, San Diego, CA) was used to assess tear film osmolarity using a technology of electrical impedance spectroscopy [23]. By using a single-use disposable chip, a nano tear sample (50 nanolitres) is collected and examined. The test chips are made of high-density injection moulded polycarbonate and are coated with a gold layer that is secure at the tip of a handheld collection pen. The inferior temporal tear meniscus is placed to collect a tiny tear sample. When the tear sample is taken, visual and audio signals are generated by the collection pen. The portable pen is then placed in the stationary scanner which analyses the tear sample by conducting an electrical signal through the tear sample and showing the osmolarity reading. Initially, we analyzed the statistical difference in tear osmolarity measurements obtained under normal (40%) and dry (5%) environmental conditions. Next, we compared the tear osmolarity measurements obtained after using Emustil at 5% (either before or after exposure to 5% relative humidity) with the osmolarity measurements obtained under 5% relative humidity. Our objective was to identify any statistical differences that may exist.

### Schirmer test

The Schirmer test was employed to measure tear production [27]. Schirmer test strip (Clement Clarke International LTD, UK) was placed into the inferior fornix sac to assess the lacrimal gland secretory function. The filter paper is wetted from tears in the fornix. The tear production can be estimated by measuring the wetting length on the strip (in millimetres) [28]. In 5 min the wetting lengths between 5 and 10 mm have been recommended as a cut-off value [29, 30].

### Phenol red thread (PRT)

This test first suggested by Hamano for measurement of tear production utilises cotton thread infused with phenol red [31]. Phenol is a pH-sensitive compound, that changes its colour from yellow to red when it is placed in the eye due to a change in the pH [32]. The thread is inserted into the lower fornix for 15 s and the tear production is estimated by measuring the length of the red (wetted) part of the thread. The main advantage of PRT over other invasive techniques like Schirmer is that it is less invasive, resulting in less reflex tearing. The endpoint reading between normal and dry eye patients is less than 10 mm in 15 s with a sensitivity of 86% and specificity of 83% [24]. PRT is more likely to measure residual tears in the lower conjunctiva rather than measure tear production [33].

The validity of tear production measurement by Schirmer test, TFI and PRT under 40 and 5% RH was assessed to see if the physical wetting properties of the Schirmer test, TFI and PRT are affected by RH. To achieve this, a pilot study was carried out to estimate the effect of room RH on these techniques. The tear production of 14 subjects (28 eyes) was measured at 40 and immediately on exposure to 5% RH using the Schirmer test, TFI and PRT. At 5% RH, the strip or thread was inserted immediately when the subject entered the dry environment to minimize ocular surface irritation by low RH or any physiological effect on production (Supplimentary file 1, Figures S1 to S3).

This pilot study showed a reduction in wetting length for all strip tests at 5% RH. The decline was statically substantial for TFI (*p = 0.001)* and PRT (*p = 0.002*) but not for the Schirmer test *(p = 0.18*). A linear regression test was applied to estimate how to convert the TFI and PRT values observed at 5% RH to the corresponding true value that would be seen at 40% RH. All data of tear production measured at 5% RH using PRT (Eq. 1) was corrected using the formula obtained from the slope of the line and intercept. The formula derived from the slope of the line was utilized to correct all tear production data measured at 5% RH using PRT. This correction aimed to mitigate the influence of dry conditions on the physical properties of the PRT and ensure that the observed changes in tear production measurements were attributable to alterations in the tear film, rather than variations in the wetting or absorption properties of the thread.

Formula to calculate PRT at 5% RH.

1 $$ {\rm{Corrected}}\,{\rm{PRT}}\,{\rm{ = }}\,{\rm{PRT}}\,\left( {{\rm{5\% }}} \right)\, * \,{\rm{0}}{\rm{.40}}\,{\rm{ + }}\,{\rm{14}}{\rm{.98}} $$

### Statistical analysis

Data was statistically analysed by PASW Statistics version 19. To examine the normal distribution of the data Kolmogorov-Smirnov test was applied. Analysis of the normally distributed data was done using repeated ANOVA measurements and post-hoc Tukey tests. Friedman’s test and the post-hoc Wilcoxon rank sum test were used to analyze data that were not distributed normally. To study the correlation between parameters Pearson’s (normally distributed) and Spearman’s (not normally distributed) tests were used.

## Results

### Tear film osmolarity

**Fig. 2:**
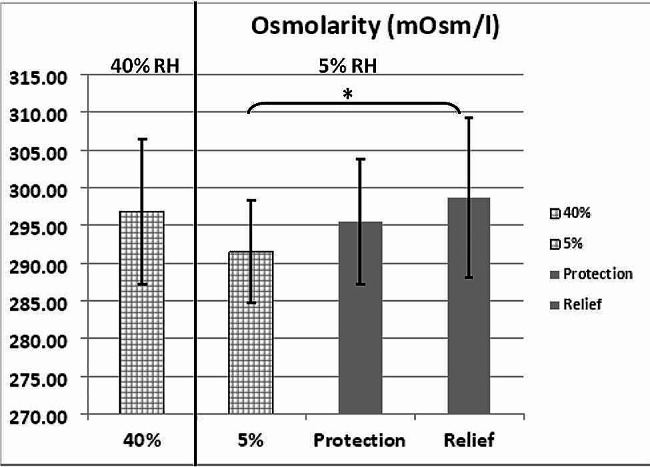
Control Tear osmolarity measurements at 40 and 5% RH (Gridded bar) and after instillation of Emustil for protection and relief. Instillation of Emustil resulted in a significant increase in tear osmolarity in the relief method compared with osmolarity seen at 5% RH when no drop was used. Pairwise significant differences are indicated by (*)

Mean tear osmolarity dropped from 296.8 mOsm/l at 40% RH to 291 mOsm/l at 5%. RH (Fig. 2). However parametric tests showed no significant variation in tear osmolarity values at 40 and 5% RH *(p = 0.084).* A significant increase in tear osmolarity was observed in relief compared to 5% with a mean value of 298 mOsm/l *(p = 0.03*) but was not statistically different to 40%. No noteworthy dissimilarity in osmolarity was observed with the use of Emustil eye drops in protection mode (295 mOsm/l) compared with both 40% and 5%.

### Tear production

**Fig. 3:**
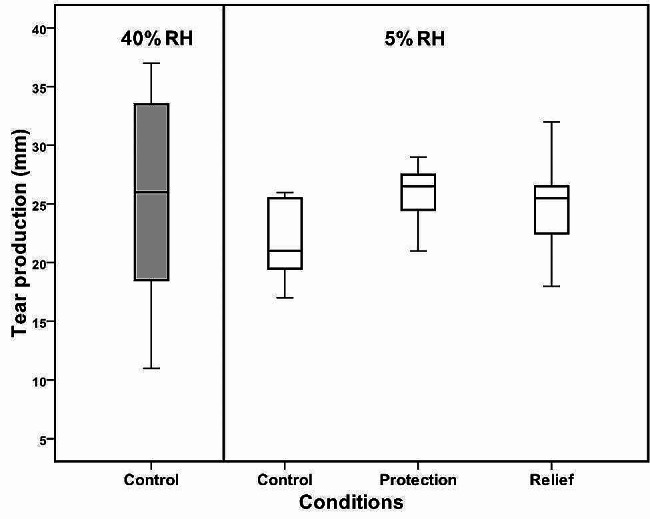
shows a large spread of PRT data obtained at 40% RH compared with 5% RH. The mean PRT value decreased from 26 ± 9 in normal conditions (40% RH) to 22 ± 4 mm in dry conditions (5% RH). However, this reduction in tear production did not reach statistical significance (*p = 0.24*). An increase in wetting length at protection and relief was seen compared with both normal and dry conditions. However, repeated measures ANOVA test showed no statistically significant differences in tear production (*p > 0.05*). The mean value of PRT was 25.7 (*p = 0.059*) in protection and 24.9 mm (*p = 0.32*) in relief

Figure 3: A box graph presenting tear production values measured without the instillation of Emustil (control) at 40 and 5% RH and with the use of Emustil in protection and relief methods (*n* = 12). No significant differences were seen in tear production. Data was corrected for observations at 5% RH caused by thread wetting at low RH. The box illustrates the interquartile limits that encompass 50% of the values. The whiskers are lines that stretch from the box to the upper and lowest measures. The line across the box signifies the median value. Pairwise significant (Tukey’s post hoc test) differences are indicated by (*).

### Ocular surface temperature (OST)

**Fig. 4:**
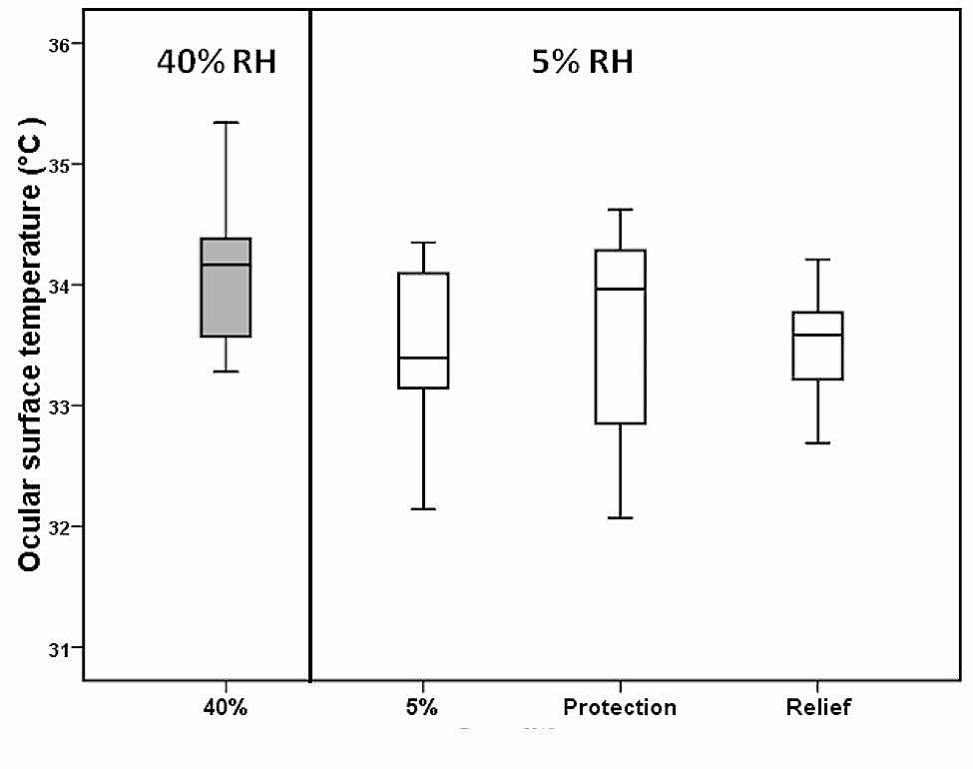
A box plot showing the ocular surface temperature at 40% and 5% without the instillation of Emustil (Control) and when the drops were instilled at different times (protection and relief). There was no difference in ocular temperature after exposure to a dry environment. Installation of Emustil did not result in a significant change in ocular surface temperature

The mean ocular surface temperature was 33.90 ± 0.89 °C at the normal environmental condition (40% RH). No significant change was found in OST following exposure to 5% RH. OST did not show a statistically significant change with the emulsion when used for relief (*p > 0.05*) (Fig. 4).

### Ocular discomfort

**Fig. 5:**
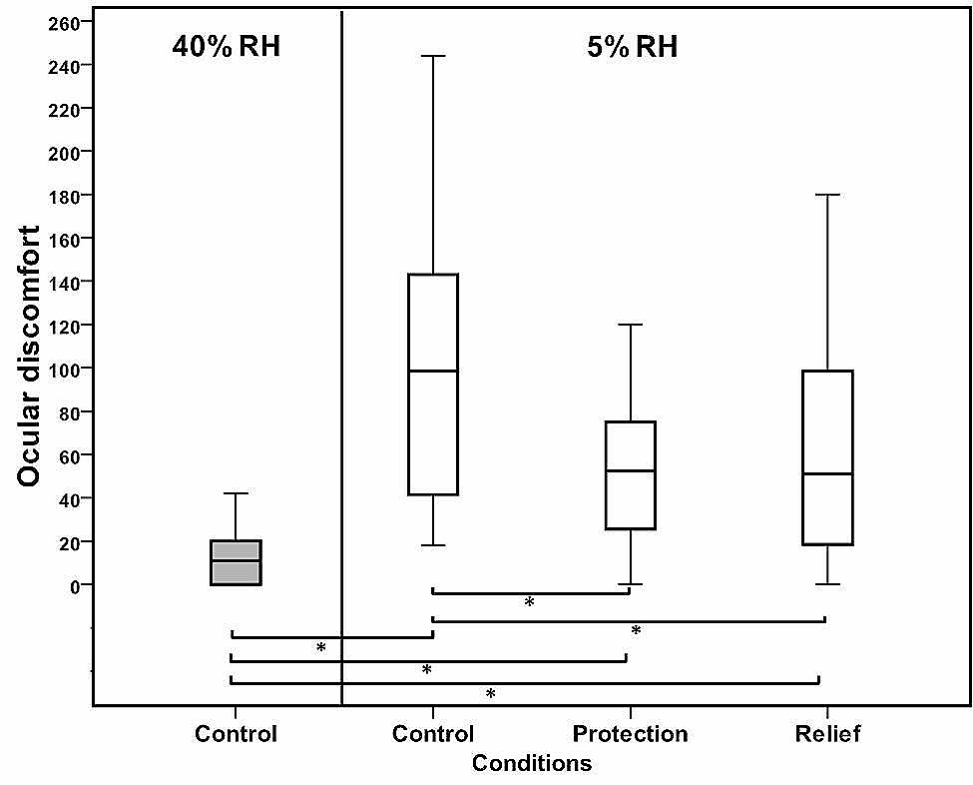
Box plot showing ocular comfort measured at 40% and 5% RH (Control) and following different treatment protocols. A significant increase was seen at 5% RH. The discomfort score was significantly improved following the instillation of emulsion for both protection and relief methods (*n* = 12). The box denotes the interquartile range that includes 50% of the values. The whiskers are lines that extend from the box to the highest and lowest values. The line across the box indicates the median value. Pairwise significant (Tukey’s post hoc test) differences are indicated by (*)

The total symptom score for ocular discomfort increased sharply when subjects were exposed to the dry environment (*p* = *0.004*). The ocular discomfort score was found to be significantly higher (*p* = *0.004*) at 5% RH compared with that observed at 40% RH even following the instillation of Emustil (Fig. 5).

However, both methods of treatment managed to improve ocular comfort in the desiccating environment. The mean score of ocular discomfort observed was 70 at 5% RH, but the instillation of the oil-in-water emulsion (Emustil) resulted in a noticeable decrease in ocular discomfort to 37 *(p = 0.00)* in protection and 59 in relief *(p = 0.05).* Although the total ocular discomfort score was lower during protection than that observed in the relief visit, this difference was insignificant *(p = 0.67)*. Pairwise differences in ocular discomfort assessed at different relative humidity are shown in Table 2.

**Table 2:** Shown in the table are Pairwise differences (Tukey’s post-hoc test) in ocular discomfort assessed 40 and 5% relative humidity before and after the use of Emustil eye drop

	5%	Protection (before)	Relief (after)
40%	0.004	0.004	0.004
5%		0.00	0.05
Protection (before)			0.67

## Discussion

Tear film osmolarity has been considered the best single test to diagnose and classify dry eye disease [34]. Despite studies indicating that osmolarity is not effective in distinguishing between dry eye types [35], tear hyperosmolarity is still one of the operative diagnostic tools for DED [36]. The tear osmolarity value in the dry eye is higher when compared with normal eyes [37]. Investigations have been done to determine the cut-off value of normal tear osmolarity. Values between 293 and 320 mOsm are the overlapping areas between normal and dry eye osmolarity [35].

To manage the signs and symptoms of dry eye, many tear film supplements have been developed with a wide range of ingredients [38]. Despite not fully curing dry eye, tear film supplements can help to reduce the signs and symptoms, and restore the ocular surface to its normal homeostatic state. Additionally, these medications prevent inflammation, reduce tear osmolarity, and substitute for tear fluid [38, 39].

To observe tear film parameters under normal (40% RH) and dry (5% RH) environmental conditions, a panel of objective and subjective measures was conducted. The use of Castor oil emulsion has previously resulted in an improvement in DE signs and symptoms [14]. The optimal time to use the medication was determined by instilling the oil-in-water emulsion drop before (protection) and after (relief) exposure to desiccating environment.

The results of previous studies agree well with the improvement in tear stability observed when using the oil-in-water emulsion [40, 41]. The oil-water emulsion’s design aims to enhance the structure and function of the lipid layer, resulting in improved tear film stability. Exposure to a desiccating environment was observed to result in a reduction in tear film osmolarity. However, this reduction in tear osmolarity did not reach statistical significance (*p = 0.084).* To see the effect of Emustil on osmolarity it is first necessary to cause a significant change in osmolarity. The sample power calculation suggests that a study involving a total of 28 subjects found a statistically significant difference in the osmolarity of tears in the test population between 40% and 5% RH (at *p* = 0.05, two-sided, power 0.8). Confirming these results may require further studies on a larger sample size. The normal homeostasis of the ocular surface could be affected by changes in tear film parameters such as evaporation rate, production, and stability of tears in an adverse dry environment. To restore normal homeostasis, a compensatory mechanism is expected to occur [34]. Increased blinking and stimulation of reflex secretion from the lacrimal and meibomian glands are part of this process [34]. Therefore, any reduction in tear osmolarity could be due to the increase in tear production and the secretion of reflex tears that are characterised by lower osmolarity when compared with basal tears [42].

An elevation of tear osmolarity was observed in both protection (295 mOsm/l, *p* = 0.29) and relief (298 mOsm/l, *p* = 0.036) compared with no therapy used (291 mOsm/l). It is noted that the mean tear osmolarity in both protection and relief increased to a value that is similar to that seen at normal humidity (296.83 mOsm/l). This indicates that the Emustil eye drop was able to contribute to the normal homeostatic status of the ocular surface during exposure to an adverse climate condition and reduce the compensatory reflex response. A previous report by McCann et al. has shown that the instillation of oil-in-water emulsion significantly improved tear osmolarity, but this was in dry eye patients and with use over 90 days [18].

The production and turnover of tears are important to protect the ocular surface against environmentally induced changes [43]. The production and turnover of tears are important to protect the ocular surface against environmentally induced changes [44]. The effect of RH on tear production strip tests has been previously observed [21, 22]. In the present study, no significant change was seen in tear production when Emustil was used under both treatment techniques (*p = 0.24)*. It should be noted that measurements of tear production depend on the absorption properties of the PRT cotton thread. The cotton thread is also exposed to 5% RH and a pilot study did show that this does affect the wetting dynamics observed. Emustil drop instillation increases lipid layer thickness and improves tear stability and therefore prevents irritation to the ocular surface and helps to stabilize lacrimal secretion levels during exposure [22]. This is in agreement with earlier findings by other investigators which showed that the use of oil-in-water emulsion for 30 days did not change tear production among dry eye patients [15].

Recently, a considerable number of studies have shown that ocular discomfort and sick building syndrome are associated with indoor workplaces characterised by low humidity [45]. In this study, an increase in ocular discomfort was seen when subjects were exposed to a desiccating environment. This is in agreement with previous work which has shown an increase in ocular symptoms among workers in dry places [46]. Furthermore, we noticed that the use of Emustil resulted in a significant enhancement in ocular comfort. Both treatment protocols managed to significantly decrease the ocular discomfort score under desiccating conditions *(p = 0.00)* and *(p = 0.05)* respectively). Previous reports have shown that an oil-in-water emulsion improved ocular comfort in dry eye patients [18, 47]. This improvement in ocular comfort in dry conditions is consistent with improvements in tear film stability and lipid layer thickness [22].

## Conclusion

This study demonstrates that the use of the CEC could provide researchers with a readily available method for quickly assessing the effectiveness of tear supplementation. In the laboratory environment, tear film symptoms that are typical for dry eye patients can be easily simulated using the CEC. This new method enables the further evaluation of tear film parameters and dry eye treatment protocols in labs before attempting them on patients with DE in clinics. Oil-in-water emulsion eye drops are effective in relieving and safeguarding tear film parameters in an adverse dry environment. A single instillation of Emustil was shown to improve tear production and ocular symptoms in ultra-dry conditions (5% RH). Furthermore, tear osmolarity and production data obtained from this study indicate that ocular homeostasis was better maintained during exposure to a desiccating environment when Emustil was used both in protection and relief.

### Electronic supplementary material

Below is the link to the electronic supplementary material.

Supplementary Material 1

## Data Availability

All the relevant data has been provided in the manuscript and supplementary files. Supplementary datasets used and/or analyzed during the current study are available from the corresponding author upon reasonable request.
