# Supplementary material for: Evaluation of therapeutic efficacy of Emustil drops for ocular discomfort and tear film osmolarity using different treatment management modes under dry environmental conditions
Source: BMC Ophthalmol. 2024 Mar 25;24:129. doi: 10.1186/s12886-024-03390-0 (PMC10962139; doi:10.1186/s12886-024-03390-0)
Supplement: Supplementary file 1 — Supplementary Material 1 [file 12886_2024_3390_MOESM1_ESM.docx]

**Title**: **Evaluation of therapeutic efficacy of Emustil drops for ocular discomfort and tear film osmolarity using different treatment management modes under dry environmental conditions**.

Ali Abusharha^1^, Ian E Pearce^2^, Tayyaba Afsar^3*^, Suhail Razak^3*^

**Affiliations**

1. Department of Optometry, College of Applied Medical Sciences, King Saud University, Saudi Arabia.
2. Glasgow Caledonian University, 70 Cowcaddence Road, Glasgow G4 0BA, UK.
3. Department of Community Health Sciences, College of Applied Medical Sciences, King Saud University, Riyadh, Saudi Arabia.

Figure S1: The effect of relative humidity on tear function index measurement. A significant reduction in wetting length of the TFI strip (p=0.001) was found as subjects (n=14) were observed in 5% RH.

**Figure S2**: **The effect of relative humidity on phenol red threads measurement. A significant reduction in wetting length of the PRT (*p=0.002*) was found as subjects (n=14) were observed in 5% RH.**

Figure S 3: Scatter plot of data obtained from phenol red thread readings at 40 and 5% relative humidity in 14 eyes.

Figure S 4: Scatter plot of data obtained from phenol red thread readings at 40 and 5% relative humidity in 28 eyes.
